# Nanopore sequencing-derived methylation biomarker prediction for methylation-specific PCR in patients with head and neck squamous cell carcinoma

**DOI:** 10.1186/s13148-025-01960-7

**Published:** 2025-09-13

**Authors:** Daria Meyer, Anne Hennig, Anna-Bawany Hums, Orlando Guntinas-Lichius, Martina Schmitz, Manja Marz

**Affiliations:** 1 RNA Bioinformatics and High-Throughput Analysis, Friedrich Schiller University Jena, Leutragraben 1, 07743 Jena, Germany; 2 oncgnostics GmbH, Löbstedter Str. 41, 07749 Jena, Germany; 3 Department of Otorhinolaryngology, Jena University Hospital, Am Klinkum 1, 07747 Jena, Germany; 4 Max Planck Institute for Evolutionary Anthropology, Deutscher Pl. 6, 04103 Leipzig, Germany; 5 German Center for Integrative Biodiversity Research (iDiv), Puschstraße 4, 04103 Leipzig, Germany; 6 Michael Stifel Center Jena, Friedrich Schiller University, Ernst-Abbe-Platz 2, 07743 Jena, Germany; 7 Cluster of Excellence “Balance of the Microverse”, Friedrich Schiller University, Fürstengraben 1, 07743 Jena, Germany

**Keywords:** Nanopore sequencing, Diagnostic biomarker, Head and neck cancer, Methylation-specific PCR, CpG islands

## Abstract

**Background:**

DNA methylation of CpG islands is altered in cancer cells. Hypermethylation of single CpG islands in the promoter regions of tumor-suppressor genes occurs already in the early stages of cancer. These methylation changes are cancer-type specific and therefore can serve as early cancer biomarker. Identifying good and reliable biomarkers is crucial for the development of diagnostic tests and their application in clinical practice and remains the most significant challenge to date.

**Results:**

Here, we present a generic workflow for the discovery and design of DNA methylation-specific PCR (MSP) biomarkers using nanopore sequencing. We show that nanopore sequencing of three control and three tumor tissue samples was sufficient to predict differentially methylated regions between head and neck squamous cell carcinoma (HNSCC) and healthy control tissue samples and to design functional MSP primers. When applied to a validation cohort of 48 HNSCC and 46 control samples, four out of six designed MSP singleplex assays achieved good sensitivity and specificity with an AUC above 0.8.

**Conclusion:**

Our resulting DNA methylation-based workflow demonstrates how long-read methylation data enable the design of adaptable, clinically relevant epigenetic assays, even with low coverage and small initial sample numbers.

## Background

DNA methylation patterns are known to be altered in cancer cells [1]. Since these changes often arise early in carcinogenesis, they hold great potential as diagnostic biomarkers [2, 3]. Although certain genomic regions have already been established as DNA methylation-based biomarkers and implemented in clinical diagnostic screening tests [4], their translation into clinical practice remains challenging, even though the number of described epigenetic signatures is rapidly increasing [5]. Head and neck squamous cell carcinoma (HNSCC) is a well-studied cancer type, and yet, over half of patients are still diagnosed at an advanced stage [6, 7], highlighting the urgent need for effective methylation-based diagnostic biomarkers. For HNSCC, differential methylation patterns are known [8, 9], but to our knowledge no methylation-based biomarker diagnostic test exists yet.

In clinical applications, quantitative methylation-specific PCR (MSP) , even though having its limitations [10], is the most widely adapted method for detecting differences in methylation and can be considered an established technique [11, 12], being both time and cost-effective [4]. For identifying suitable MSP regions, Illumina HM450 (~450 000 CpGs) and EPIC BeadChips (~850 000 CpGs) are widely used for genome-wide DNA methylation studies [13–16]. An alternative to array-based methods, which cover only about 3% of all human CpGs [17], is whole genome bisulfite sequencing (WGBS) [18], which allows analysis of CpG sites throughout the genome, but comes with the cost of amplification bias [19]. A technology that has emerged in the last decade is nanopore sequencing, which provides per-base 5mC DNA methylation data throughout the whole genome with the additional advantage of bypassing the need for bisulfite conversion and amplification [20].

In this work, we present a generic approach to identify clinically relevant MSP primer regions for specific cancer types using nanopore sequencing across the entire human genome. These primers can then be applied via MSP as a cost-effective diagnostic tool for individual patients. We established MSP primers based on three HNSCC and three control nanopore sequenced samples and validated the approach on 48 HNSCC and 46 control tissue samples. Five out of six markers showed significant differences in MSP $\Delta$C$_T$ values between HNSCC and control samples, and four out of six markers achieved an area under the curve (AUC) greater than 0.8.

## Material and methods

### Control and tumor tissue samples

Tissue samples, including both tumorous and control specimens, were obtained from 100 patients at Jena University Hospital. The use of individual tissue samples in this study was approved by the local ethics committee (number 2019–1552) and conducted according to the principles of the Declaration of Helsinki. All information regarding the human tissue samples were managed using anonymous numerical codes. Primary tumor tissue was excised from HNSCC patients during standard surgery as part of the therapy, as given in Table S1. Control samples, consisting of mainly uvula, tonsil, and larynx tissue were collected during surgery from control patients without malignant diseases. All tissue samples were collected during surgical procedures, cryopreserved, and stored at − 80 $^\circ$C until DNA extraction. Of the twelve 10 $\upmu$m tissue sections, the first and last were used for hematoxylin and eosin (H&E) staining to assess tumor cell content based on microscopic evaluation, while the intermediate sections were used for genomic DNA (gDNA) isolation. DNA was extracted using the Macherey–Nagel NucleoSpin® Tissue Kit, following the manufacturer’s protocol [21]. The human papillomavirus (HPV) status of all samples was determined using a HPV16/18 PCR-enzyme immunoassay (PCR EIA) [22] from gDNA. The samples were analyzed for HPV16, HPV18, and two HPV-type mixtures: mix1 (HPV 31, 33, 35, 39, 45, 51, 52, 56, 58, 59, and 68) and mix2 (HPV 26, 53, 66, 67, 70, 73, and 82).

Of the 100 tissue samples, six were selected for nanopore sequencing to identify MSP primers—three HNSCC samples (T-0044-C, T-0085-C, T-0126-C) and three control samples (T-0025-N, T-0045-N, T-0099-N). The three tumor samples were selected for high tumor percentage, identical localization (oral cavity), and same sex (male). The control samples were selected for matching sex and matching similar localization (uvula, i.e., oral cavity epithelial tissue). The remaining 94 tissue samples were used for validation with methylation-specific PCR. This validation cohort was more diverse in terms of sex, age, tumor localization, and HPV status, as given in Table 1.

**Table 1:** Patient characteristics for 48 HNSCC and 46 control samples, which were analyzed with the MSP assay. Tumor classification is based on the TNM staging system (8th edition) describing tumor size (T), the occurrence of cancer cells in lymph nodes (N), and metastasis (M). Data for nanopore sequenced samples are excluded here, but shown in Table 2. A more detailed overview of all control and HNSCC samples is given in Table S1

	HNSCC ( *n* = 48)	Control (*n* = 46)
Age in years: mean (range)	62 (34–82)	44 (18–79)
Sex: male + female	38 + 10	31 + 15
Tumor localization:		
Oral cavity	17	
Tonsil	7	
Oropharynx	3	
Larynx	11	
Hypopharynx	10	
HPV status: pos + neg	8 + 40	
TNM stage (8th edition):		
T1/T2/T3/T4	7/12/13/16	
N0/N1/N2/N3/NX	19/10/11/7/1	
M0/M1	45/3	

### Nanopore sequencing with adaptive sampling

To gain per-base 5mC DNA methylation information throughout the whole genome, direct DNA nanopore sequencing was performed according to the manufacturer’s protocol (by Oxdord Nanopore Technologies). 1500 ng of the extracted DNA per sample was used as starting material for library preparation with LSK109. All samples were sequenced on R9.4.1 flow cells on a MinION Mk1B sequencing device, under minKNOW (minKNOW core v4.3.4 and v5.3.1). During sequencing, one or multiple wash steps were performed (see Table 2), using the Flow Cell Wash Kit (EXP-WSH003) to increase the sequencing yield, for all samples except T-0045-N on flow cell FAS60674. For washed flow cells, the library was split before sequencing, and loaded partially in the beginning of the sequencing run, and after each wash step.

Adaptive sampling [23] was performed to enrich the sequencing depth on the CpG islands. The CpG island annotation was retrieved from the UCSC genome browser [24], which lists 31,144 CpG islands [25]. The annotated CpG islands range in length from 200 to 45,712 nt with an average length of 777 nt and an average GC content of 67%. During sequencing with adaptive sampling, the data were live basecalled and mapped to the hg38 reference genome [26] in MinKNOW. For adaptive sampling, the CpG island regions of interest were extended for 2,000 nt on each site as "buffer" region as advised by ONT. For the complete list, as given in supplemental material.

If sequencing resulted in less than 10 X sequencing depth on the CpG islands, the sample was sequenced again, on an additional Flow Cell, as given in Table 2.

### Basecalling and methylation calling

After sequencing, the raw data was re-basecalled using the ONT provided basecaller Guppy (v6.5.7+ca6d6af), available to ONT customers via the ONT community site (https://community.nanoporetech.com), with the model dna_r9.4.1_450bps_modbases_5mc_cg_sup.cfg for calling modified cytosine bases during sequencing with the parameters –device cuda:0, for working on the GPU, and –disable_qscore_filtering to not filter reads based on quality score. Afterward, the sequencing depth for the entire genome and for the CpG islands was determined using mosdepth (v0.3.3) [27].

### MSP region prediction via diffMONT

The bam files resulting from basecalling were converted into bedmethyl files using modbam2bed (v0.9.4) developed by ONT.^1^ Differentially methylated regions applicable for MSP were predicted using diffMONT (v1.0) [28]. This tool predicts potential primer regions with at least three CpGs within 24 nt, which are methylated in at least one tumor sample, while being unmethylated (below a specified threshold) across all control samples. If two such regions appear within a given range, they are scored based on sequencing depth and methylation in the tumor samples. DiffMONT was run with default parameters (–minCpGs 3, –maxMethControl 10, –minPrimerLength 18, –maxPrimerLength 24, –minAmpliconLength 60, –maxAmpliconLength 400).

### Primer and probe design

Primers for bisulfite PCR are designed based on the regions predicted by diffMONT (±200 bp), with a length of 14-22 nt. The resulting amplicons range from 80 to 400 bp. To ensure effective methylation detection, the amplicons contain at least three methylated CpG dinucleotides and ideally four unmethylated cytosines, with a GC content of approximately 60%. Primers are designed to preferably end on a cytosine nucleotide, ensuring a melting temperature difference of < 5 $^\circ$C. The design minimizes dimer formation, avoids secondary structures, and ensures low complementarity at the 3’ end to prevent cross-primer dimers.

Probes are designed to have an ideal GC content of 50% and a melting temperature of 5–10 $^\circ$C higher than that of the primers. Their length ranges from 20 to 30 nt, and they are positioned close to the 3’ end of the forward primer while avoiding a guanine at the 5’ end and palindromic sequences. Additionally, care is taken to prevent the formation of secondary structures with themselves or the primers.

Primers and probes are synthesized by Eurofins Genomics [29]; probes are labeled with FAM fluorescent dye.

### Analytical evaluation of primers and probes

The performance of methylation-specific primers is tested using a mastermix with hot-start polymerases with an intercalating dye for primer analysis and melt curve analysis without fluorescent oligonucleotide probes, running 43 PCR cycles. Primer and probe optimization is performed using gradient PCR and sensitivity dilution series. Analytical sensitivity is evaluated using template DNA amount of 10 ng, 1 ng, and 0.1 ng, while analytical specificity is assessed using positive controls (*in vitro* methylated and bisulfite-treated DNA), negative controls (50 ng whole-genome amplified DNA), and no-template controls (water). To determine the optimal hybridization temperature, a gradient PCR with temperatures ranging from 55 to 68 $^\circ$C is performed. Evaluations are based on C$_T$ values, primer dimer formation, and melting curve analysis.

Primers and probes are excluded if they produce false-negative results due to inefficient annealing, resulting in lack of or late amplification. Another reason for exclusion are false-positive results due to nonspecific binding, leading to unspecific amplification in negative or no-template controls. All primer and probe sequences are available upon request.

### Bisulfite conversion

Bisulfite conversion was applied to 150 ng gDNA, using the EZ DNA Methylation-Gold Kit provided by Zymo Research [30], following the manufacturer’s protocol. This treatment selectively deaminates unmethylated cytosines to uracils, while methylated cytosines remain unchanged, thereby preserving the DNA methylation pattern. The bisulfite-modified DNA served as the template for MSP. Specific primer sets targeting methylated of the region of interest were used to discriminate between methylated and non-methylated alleles. The presence of methylation markers enabled the identification of malignant or premalignant cell populations. An internal control assay was included to assess the quality and quantity of the bisulfite-converted DNA and to ensure successful amplification.

### Quantitative methylation-specific PCR (MSP)

To assess DNA methylation levels and oligonucleotide performance, quantitative real-time PCR (qPCR) was done on bisulfite-modified DNA and control DNA. Thermal cycling using the CFX96 Touch Real-Time PCR Detection System (Bio-Rad Laboratories, Inc.) started with a denaturation step at 94 $^\circ$C for 1 min followed by 43 cycles at 94 $^\circ$C for 15 sec and the designated annealing temperature $^\circ$C for 30 sec ending with cool down for 1 min at 30 $^\circ$C. The cycle threshold (C$_T$) value is the point where the amplification curve intersects the threshold line, serving as a relative measure of the target concentration in the PCR. For each marker, the threshold was set at the beginning of the exponential phase of the amplification curve when using a linear scale. The relative methylation level of a sample was determined by assessing the ratio of the target gene C$_T$ value to the bisulfite-specific reference primers targeting ACTB (beta-actin, 133 bp) C$_T$ value. The delta between the target gene and ACTB ($\Delta$C$_T$) was calculated to account for variations in DNA input amount and quality. By normalizing the target signal to the reference signal, $\Delta$C$_T$ provided a relative quantification of DNA methylation, reducing technical biases and enabling accurate sample comparisons.

### Data visualization and statistical analysis

For IGV snapshots in Figs. 3 and S2, the Integrative Genomics Viewer (IGV) (version snapshot 05/04/2023 11:31) [31] was used. Alignments were colored by 5mC methylation, insertion marks were removed, and small indel threshold was set to 10, and reads are shown as "collapsed". The *p* values in Fig. 6 were calculated in R (v2022.07.0+548) [32] using the function t_test from the package rstatix using Bonferroni correction [33] to adjust for multiple testing. Given the limited number of tests in the scenarios considered in this work, Bonferroni correction provides a conservative and widely accepted method for controlling the family-wise error rate [34]. Optimal $\Delta$C$_T$ value thresholds for classification into HNSCC and control were calculated using the library cutpointr (v1.2.0) [35] in R. Thresholds were selected by maximizing the Youden Index, which is defined as $J = \textrm{sensitivity} + \textrm{specificity} - 1$ and is a well-established criterion for measuring the performance of diagnostic tests [36, 37].

## Results

### A general workflow for methylation marker discovery in cancer

**Fig. 1:**
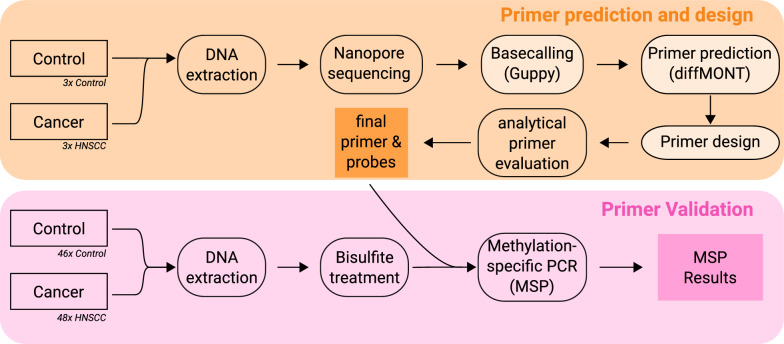
Based on nanopore sequencing data of three HNSCC and three control tissue samples, MSP primers were designed, and analytically evaluated. The best six primer pairs were chosen and validated with MSP assays for bisulfite-treated DNA extracted from 48 HNSCC and 46 control tissue samples. Steps, which were done purely *in silico*, are shown in a brighter orange (Basecalling, Primer prediction, Primer design)

We developed a two-step, genome-wide workflow for the identification and validation of cancer-specific DNA methylation markers, as shown in Fig. 1. This approach is designed to be broadly applicable to a variety of cancer types and enables the systematic discovery of candidate regions with differential methylation patterns suitable for downstream MSP analysis.

***Step 1: Marker discovery and primer design using nanopore sequencing*** This initial phase is relatively cost-intensive and involves sequencing a small number of cancer and control tissue samples using Oxford Nanopore Technologies (ONT) for the purpose of primer prediction and design. We recommend at least three cancer and three control samples, each sequenced on at least one ONT MinION flow cell. Genomic DNA is extracted from fresh-frozen tissue, and each sample is sequenced on at least one ONT flow cell. Methylation profiles are then analyzed using our in-house computational tool, diffMONT, which identifies differentially methylated regions (DMRs) across the genome and predicts suitable MSP primers for downstream primer validation. The overall cost for this discovery phase, including DNA extraction, library preparation, and sequencing, is currently estimated at approximately €7000.

***Step 2: Validation by methylation-specific PCR*** The second phase of the workflow is designed for cost-effective validation of candidate markers in a larger patient cohort. This step requires standard molecular biology equipment and reagents. DNA is extracted from an extended set of cancer and control samples (we recommend at least 50 in total), followed by bisulfite conversion and methylation-specific PCR using the primers predicted in step one. The validated primers then allow for rapid screening of potential biomarkers at a low cost of less than €10 per sample and can be scaled depending on the availability of samples and the number of regions to be tested.

To demonstrate the feasibility and versatility of this approach, we applied the workflow to HNSCC samples. From the 100 available tissue samples, three HNSCC and three control samples were selected for nanopore sequencing and subsequent primer prediction. The remaining 94 samples, which included a diverse cohort in terms of sex, age, and tumor localization, were used for validation with MSP. The following sections detail each step of the workflow in the context of HNSCC, while the same principles may be transferred to other tumor types with minimal adaptation.

### Primer prediction and design for HNSCC markers

***Nanopore sequencing dataset results in 10 X sequencing depth on CpG islands with a tumor cell percentage of 70–80%***

For nanopore sequencing and *in silico* marker prediction, three male HNSCC tissue samples with a high tumor cell percentage (T-0044-C, T-0085-C, T-0126-C) and three male control samples (T-0025-N, T-0045-N, T-0099-N) were chosen, as given in Table S1 and Fig. S1. For all tissue samples, genomic DNA could be extracted successfully. One of the six samples (T-0044-C) was tested positive for HPV 16.

To achieve a sequencing depth of 10 X on the CpG islands—which has been demonstrated as sufficient for reliable DNA methylation analysis using nanopore sequencing [38, 39]—adaptive sampling was applied to selectively enrich for sequencing depth on CpG islands. We focused on CpG islands, which account for $\sim$2% of the human genome [40], as they contain many of the regulatory methylation sites found in the genome [41]. Sequencing of the individual flow cells resulted in 4.11 Gb (T-0045-N) to 24.82 Gb (T-0025-N) output, which after basecalling resulted in a whole-genome coverage between 4.65 X and 8.44 X. Finally, for the CGI regions of each sample we gained a robust sequencing depth between 11.98 X (T-0126-C) and 28.66 X (T-0025-N), as given in Table 2.

In summary, we end up with a sufficiently reliable dataset for detecting differentially methylated regions with a tumor cell percentage above 70 % in the HNSCC samples, and a sequencing depth of at least 10 X on CpG islands.

**Table 2:** Characteristics and results of nanopore sequenced samples.

		Sample cCharacteristics					Sequencing Run Stats					Sample Stats	
	Sample	Sex	Age	TC	Size	TNM	Wash	AS	Yield	HSA	CGI	HSA	CGI
			[years]	[%]	[cm$^2$]	stage			[Gb]	[X]	[X]	[X]	[X]
Control	T-0025-N	m	59	0	0.16		1x	Yes	6.46	1.74	5.40	8.44	28.66
							1x	Yes	24.82	6.70	23.26		
	T-0045-N	m	22	0	0.50		1x	Yes*	5.45	2.35	3.52	7.83	18.07
							0x	Yes	4.11	1.06	2.81		
							1x	Yes	16.11	4.42	11.74		
	T-0099-N	m	57	0	1.50		1x	Yes	24.40	6.03	16.74	6.03	16.74
HNSCC	T-0044-C	m	53	80	0.91	T3N0M0	2x	No	13.49	3.92	4.20	6.53	13.35
							1x	Yes	9.68	2.61	9.15		
	T-0085-C	m	74	80	0.20	T4N1M0	1x	Yes	17.13	4.65	14.63	4.65	14.63
	T-0126-C	m	59	70	0.25	T4N0M0	1x	Yes	23.50	5.98	11.98	5.98	11.98

**Sample Characteristics:*** TC* Tumor cell percentage was determined based on hematoxylin and eosin (H&E)-stained slides; Size—Tissue section size is given in cm$^2$.

**Sequencing Run Stats:** Wash—number of times the Flow Cell was washed and reloaded with the same sample; AS—Adaptive Sampling applied in minKNOW (Yes*—AS was applied after washing); Yield—sequencing output in gigabases (Gb);HSA—sequencing depth on the entire genome per flow cell; CGI—sequencing depth on the CpG island only per flow cell. **Sample Stats:** HSA—Sequencing depth per sample for the whole genome (HSA); CGI—Sequencing depth per sample for CpG islands (CGI). Each line of the table refers to one flow cell

***In silico predicted regions are distributed over all chromosomes and overlap with both CpG islands and annotated genes***

For the identification of potential epigenetic biomarker regions throughout the human genome, diffMONT was applied on the six nanopore sequenced samples to screen for hypermethylation in MSP-suitable regions in the three HNSCC tissue samples in comparison to the three control tissue samples.

**Fig. 2:**
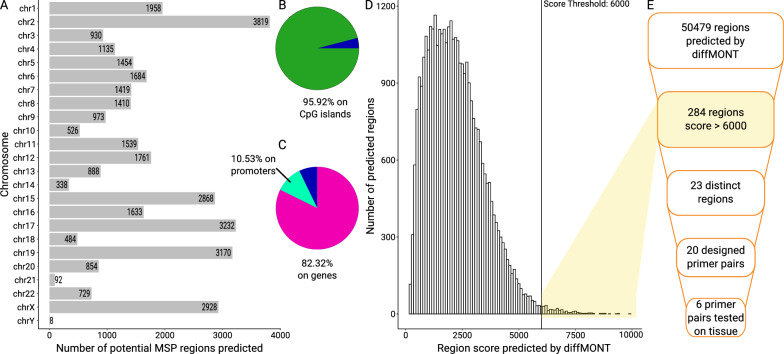
DiffMONT predicted a total of 50,479 regions. **A** The regions are distributed unevenly among the chromosomes. Data for 14,647 predicted regions located on unassembled chromosome patches are not shown. **B** 95.92% (48,420) of the predicted regions overlap with CpG islands. **C** 82.32% of the predicted regions overlap with annotated genes, 10.53% overlap not with genes but with promoters (500 nt upstream of transcription start site), and 7.15% with neither gene nor promoter. **D** Score distribution for the predicted regions. Higher scores result from high tumor sample methylation and coverage. 284 predicted regions have a score above 6000. **E** Following the diffMONT predictions, a manual selection process was conducted to determine the final primer choice

DiffMONT reported in total 50,479 regions distributed over all chromosomes, as shown in Fig. 2A. Of the predicted regions, 95.92% (48,420/50,479) overlap with annotated CpG islands in the genome, as shown in Fig. 2B. A high overlap with CpG islands was expected, because i) many regulatory methylation sites are located on CpG islands [41], and ii) sequencing was enriched for CpG islands. Of the 50,479 predicted regions, 82.32% (41,556/50,479) overlap with annotated genes, as shown in Fig. 2C. Each predicted region was scored by diffMONT based on methylation and coverage in the HNSCC samples, whereas a higher score indicates higher methylation and coverage. The score ranges from 203.98 to 9937.82 for the predicted regions, as shown in Fig. 2D. For the further analysis, only the top 284 regions with a score above 6000 were considered, as shown in Fig. 2E.

***All high-scored predicted regions overlap with CpG islands but are in different genomic contexts—some overlap known HNSCC genes***

Many of the individual MSP regions predicted by diffMONT are located very close to each other and have similar scores. This is expected, since we enriched for CpG islands, and cytosines throughout one CpG island are generally homogeneously methylated [42]. As a result, many of the predicted MSP regions overlap each other. To identify the most suitable primer sites for cancer detection, we selected only the highest-scoring MSP region from each overlapping cluster.

After this filtering step, 23 distinct MSP regions with a score > 6000 remained, which are distributed across 13 chromosomes, and all located on CpG islands, as given in Table S2.

CpG islands are often described in the context of gene promoters, however about half the CpG islands are not associated with annotated promoters [43]. Instead, they can be located upstream of genes, in exons, introns, downstream of genes, as well as intergenic. Similarly, our predicted MSP regions are distributed across the genome, found both in association with genes and in intergenic regions.

**Fig. 3:**
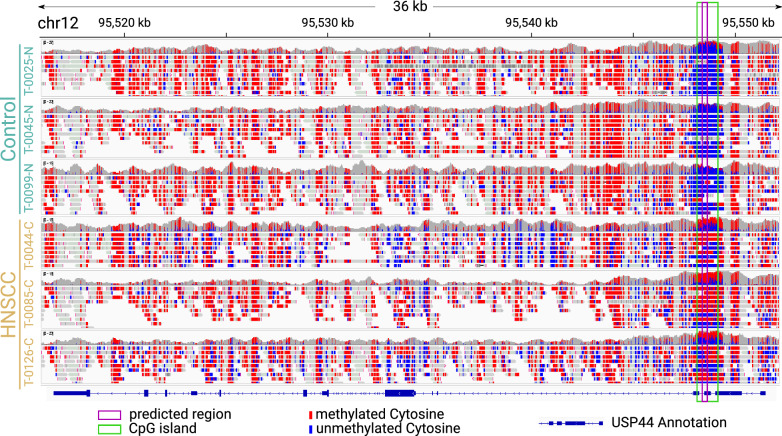
The highest-scored predicted MSP region (204 nt, see also Table S2) is located in a CpG island on USP44

The highest-scored predicted region is located on a CpG island and overlaps both an exon and intron of the annotated gene USP44 (Ubiquitin carboxyl-terminal hydrolase 44), as shown in Fig. 3. This finding is supported by previously reports of the USP44 promoter described to be hypermethylated in nasopharyngeal carcinomas [44]. As expected, all reads from the control samples have a conserved low methylation in the predicted region and the reads from HNSCC samples are highly methylated.

Sample T-0085-C shows a slightly higher percentage of methylated reads compared to samples T-0044-C and T-0126-C, even though their total tumor cell content is similar. Interestingly, in the genomic context of the CpG islands the methylation pattern changes abruptly, and all control samples as well as the HNSCC samples show high methylation.

Another predicted region overlaps the bidirectional promoter of the genes ZNF529 and ZNF382, as shown in Fig. S2A. Methylation differences in bidirectional promoters have a double meaning in the context of gene expression regulation [45]. Foy et al. list promoter methylation of ZNF529 as potential marker candidate for OSCC [46].

In contrast, some of the predicted regions do not overlap with any annotated genes, as shown for the predicted region on chromosome 20, as shown in Fig. S2B.

Another 15 genes are overlapping with the the 23 highest-scored MSP regions, as given in Table S2. Among them, FLI1 was used by Gissi et al. for screening HNSCC based on DNA methylation in oral brushing samples [47].

***Primer design based on in silico predicted regions***

For the 23 distinct MSP regions, as given in Table S2, the goal was to design primers for at least one contiguous CpG island per chromosome, ensuring that the primer binding sites cover the target regions as precisely as possible and include at least three predicted CpG sites. For optimal design, however, it was necessary to adjust the primer boundaries by approximately ±5 nucleotides to optimize for the *in silico* calculated melting temperature (Tm). Of the 23 MSP regions, a subset was taken for further analysis, due to limited resources. The subset was selected by sorting the 23 regions by score, and taking the top 14 regions. Additionally, two regions on previously unrepresented chromosomes were added. As no primer design was possible for the selected region on chromosome 8, the next highest-scoring region on chromosome 8 was chosen. This selection resulted in 16 regions, for which primer candidates were selected for potential methylation-specific primer and probe design, resulting in 20 primer combinations, as given in Table S3. In the following, the designed primer pairs will be named based on the closest annotated gene, followed by PP (Primer Pair) and the score of the corresponding predicted MSP region (superscript), e.g., FLI1-PP1$^{7990}$. If multiple primers were designed for a close related region, they were numbered consecutively (PP1, PP2,...).

***Analytical evaluation of primer performance*** To determine the optimal annealing temperature for each primer pair a gradient PCR was performed as an initial step. Primer pairs were evaluated based on their amplification performance, characterized by a sigmoidal amplification curve, specific target amplification, low cycle threshold (C$_T$) values, minimal primer dimer formation, and melting curve profiles without unspecific products in negative controls (NC) and no-template controls (NTC).

***Gradient PCR and confirmation of annealing temperature in primers***

Out of 20 primer pairs tested, two primer pairs (FLI1-PP2$^{7990}$ and PER1-PP1$^{6931}$) were excluded due to non-specific amplification. The primer pairs USP44-PP1$^{9938}$ and LINC02579-PP1$^{7654}$ were excluded due to non or inefficient amplification with high C$_T$ values, respectively, indicating insufficient analytical sensitivity across the tested temperature range, as given in Table S3.

Amplification profiles allowed for the selection of optimal annealing temperatures for 16 primer pairs for further testing (RAI1-PP1$^{6789}$, USP44-PP3$^{9937}$, USP44-PP4$^{6366}$, FAM43A-PP1$^{6113}$, KREMEN2-PP1$^{6594}$, BEND4-PP1$^{6417}$, KCNB1-PP1$^{7427}$, SMDP5-PP1$^{6131}$, RAI1-PP2$^{6789}$, EMX1-PP1$^{6283}$, KCNB1-PP2$^{6707}$, ZNF529-PP1$^{7480}$, LHX8-PP1$^{6009}$, URAD-PP1$^{6247}$, FLI1-PP1$^{7990}$, and USP44-PP2$^{9938}$).

The primer pair RAI1-PP2$^{6789}$ was selected for further analyses, as both primer sets (RAI1-PP1$^{6789}$ and RAI1-PP2$^{6789}$) showed comparable performance and targeted a similar predicted genomic region.

Following the gradient PCR, the previously determined annealing temperatures for all selected primer pairs were validated in a subsequent experiment. This involved amplification using a dilution series of the positive control (PC), including 10 ng and 1 ng input, alongside negative controls (NC) and no-template controls (NTC). We confirmed the analytical sensitivity of each primer pair and validated the annealing temperatures on the qPCR instrument by reproducing the C$_T$ values observed during the gradient PCR, as given in Table S3.

***Probe design and confirmation of probe functionality*** Additionally, for each of the validated amplicons, probes were designed. In total, 15 probes were designed for the most promising primer candidates (USP44-PP3$^{9937}$, USP44-PP4$^{6366}$, FAM43A-PP1$^{6113}$, KREMEN2-PP1$^{6594}$, BEND4-PP1$^{6417}$, KCNB1-PP1$^{7427}$, SMDP5-PP1$^{6131}$, RAI1-PP2$^{6789}$, EMX1-PP1$^{6283}$, KCNB1-PP2$^{6707}$, ZNF529-PP1$^{7480}$, LHX8-PP1$^{6009}$, URAD-PP1$^{6247}$, FLI1-PP1$^{7990}$, and USP44-PP2$^{9938}$). To evaluate probe performance, an initial gradient PCR (PC, NC, NTC as template) was again performed to determine the optimal annealing temperature for downstream applications and to assess probe functionality. Ideally, the C$_T$ values obtained with the probe-based assays were expected to closely match those from the corresponding primer reactions with intercalating dye, indicating efficient probe hybridization and amplification without compromising assay sensitivity.

Of the 15 designed probes, an optimal annealing temperature could be directly established for 13 primer-probe combinations based on their amplification profiles. For USP44-PP3$^{9937}$ and USP44-PP4$^{6366}$, no probe signal was detected in the positive control when using the respective probes, as shown in Fig. S3. As a result, USP44-PP3$^{9937}$ and USP44-PP4$^{6366}$ were rejected.

**Fig. 4:**
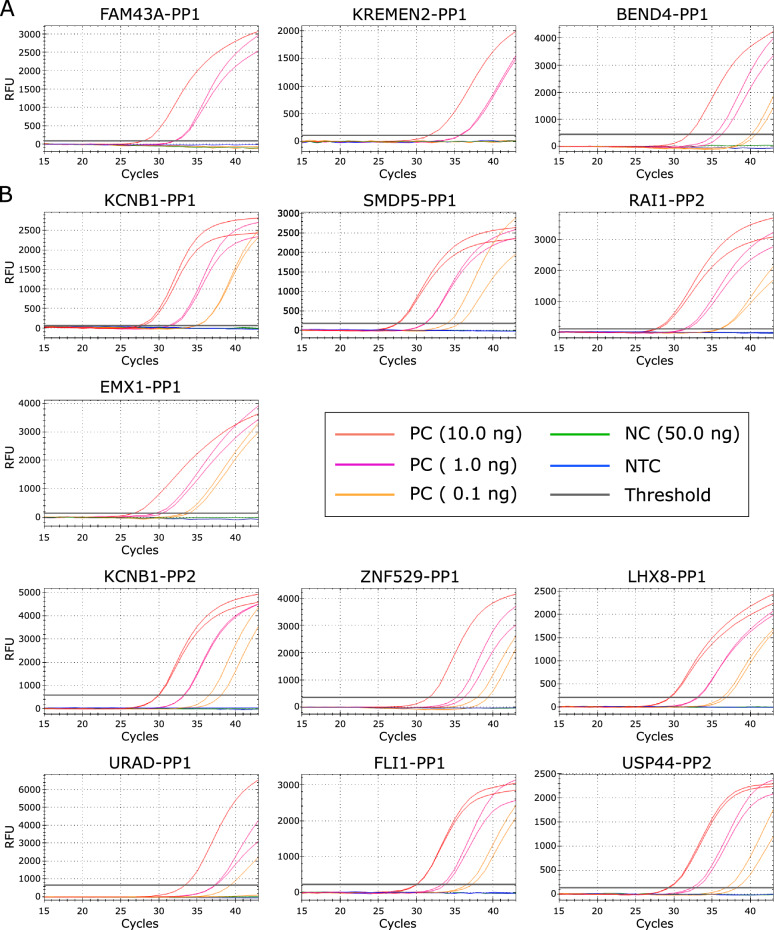
**A** Amplification curves of primer probe combinations show no probe binding in FAM43A-PP1$^{6113}$ and KREMEN2-PP1$^{6594}$ for 0.1 ng PC. BEND4-PP1$^{6417}$ shows increased C$_T$ values, especially for 0.1 ng PC. **B** All other primer probe combinations show specific amplification curves for all three dilutions. The curves illustrate high assay sensitivity, with consistent amplification of the target in the positive control, as well as high assay specificity, with no amplification in the negative controls. RFU—shows the resulting fluorescence signal in relative fluorescence units (RFU)

Building on this, the probe performance was evaluated by examining the amplification curves for a dilution series of the template (10 ng, 1 ng, 0.1 ng PC), as shown in Fig. 4. This step served to confirm both the annealing temperature settings and the analytical sensitivity of each assay. These assays demonstrated C$_T$ values closely matching those obtained in the corresponding primer-only reactions, indicating that probe integration did not compromise assay performance and sensitivity, as given in Table S3.

Both FAM43A-PP1$^{6113}$ and KREMEN2-PP1$^{6594}$ show no amplification curve for 0.1 ng of input template. BEND4-PP1$^{6417}$ showed increased C$_T$ values especially for 0.1 ng of input template. Therewith, amplification is no longer reliably detectable. Consequently, these three primer-probe combinations were excluded from further analysis, as shown in Fig. 4A. The remaining ten primer probe combinations performed well in the dilution series PCR, as shown in Fig. 4B.

In the next step, a selection was made regarding which primer-probe combinations would be used for further clinical validation. Although ten assays demonstrated robust performance, the choice was guided by a cost-effective use of available tissue material. Six candidate markers were selected (KCNB1-PP2$^{6707}$, ZNF529-PP1$^{7480}$, LHX8-PP1$^{6009}$, URAD-PP1$^{6247}$, FLI1-PP1$^{7990}$, and USP44-PP2$^{9938}$) based on their strong analytical performance and promising potential according to preliminary scoring results. The inclusion of additional markers remains a possibility for future analyses.

### Validation of designed primers with MSPs demonstrates diagnostic potential in HNSCC and controls

***First clinical validation of nanopore-derived primer pairs by MSP***

After *in silico* prediction and analytical evaluation of the six primer pairs, their diagnostic utility was first tested in MSP assays for the three control and three HNSCC samples used for nanopore sequencing.

As expected, for each of the six regions, the MSP assay demonstrated higher $\Delta$C$_T$ values for the control samples compared to the HNSCC samples, as given in Table 3. Despite a notable range of variability observed within both control and tumor samples (reaching up to 10.9 cycles for ZNF529-PP1$^{7480}$ in control samples, and up to 19.62 for KCNB1-PP2$^{6707}$ HNSCC samples), the minimum of $\Delta$C$_T$ values from control samples was always higher than the maximum of HNSCC samples, with the notable exception of KCNB1-PP2$^{6707}$ in patient T-0126-C.

**Table 3:** MSP $\Delta$C$_T$ values for the predicted biomarker regions showed lower values in the HNSCC samples compared to the control samples for FLI1-PP1$^{7990}$, LHX8-PP1$^{6009}$, USP44-PP2$^{9938}$, ZNF529-PP1$^{7480}$, and URAD-PP1$^{6247}$. The only exception was a high $\Delta$C$_T$ for KCNB1-PP2$^{6707}$ in sample T-0126-C

	Sample	FLI1	LHX8	USP44	KCNB1	ZNF529	URAD
Control	T-0025-N	20.82	8.01	9.29	12.87	20.82	4.71
	T-0045-N	19.03	16.40	9.74	19.03	13.85	8.08
	T-0099-N	11.93	7.13	8.76	9.34	9.92	9.45
HNSCC	T-0044-C	0.73	0.46	1.24	0.18	3.41	1.97
	T-0085-C	2.91	1.56	2.42	2.58	4.98	1.75
	T-0126-C	7.16	1.83	7.14	19.80	4.02	2.09

**Fig. 5:**
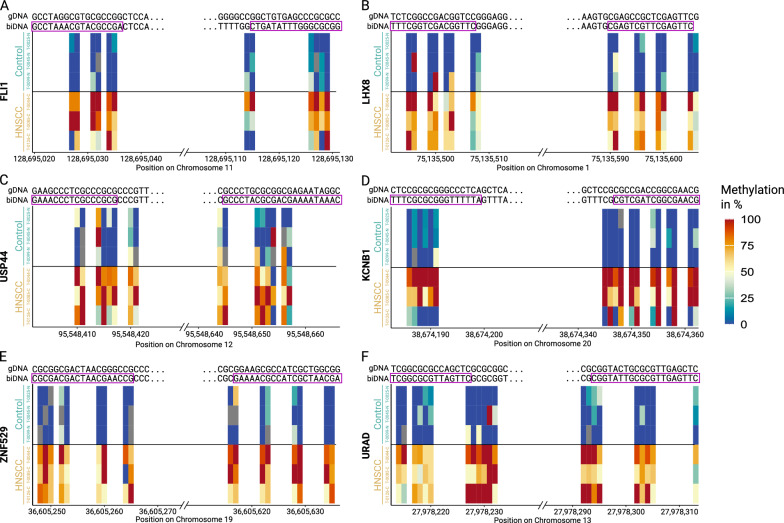
Average 5mC methylation resulting from nanopore sequencing of three control (turquise, top) and three HNSCC (orange, bottom) samples shows high methylation in HNSCC and low methylation in control samples for the final MSP primer regions FLI1-PP1$^{7990}$, LHX8-PP1$^{6009}$, USP44-PP2$^{9938}$, KCNB1-PP2$^{6707}$, ZNF529-PP1$^{7480}$, and URAD-PP1$^{6247}$. The gDNA and biDNA sequences are shown for the sense strand in 5’$\rightarrow$3’ direction. The average methylation per cytosine is strand-specific

To compare our MSP primer $\Delta$ C$_T$ values with nanopore sequencing data and to assess individual patient-level methylation patterns, Fig. 5 illustrates the average 5mC methylation in the three HNSCC and three control samples, showing high methylation in HNSCC and low methylation in control samples across the final MSP primer regions—FLI1-PP1$^{7990}$, LHX8-PP1$^{6009}$, USP44-PP2$^{9938}$, KCNB1-PP2$^{6707}$, ZNF529-PP1$^{7480}$, and URAD-PP1$^{6247}$. The nanopore sequencing and MSP confirm the differential methylation for the regions detected by nanopore sequencing within these six samples. In case of primer pair KCNB1-PP2$^{6707}$, the particular patient T-0126-C indeed has reduced methylation, comparable to control samples, as shown in Fig. 5D. Thus for these six samples, nanopore sequencing confirms the MSP results.

***Selected primer pairs show diagnostic potential in clinical OSCC samples***

Next, the validated primers were applied on additional clinical HNSCC tissue sample. As the nanopore sequenced samples used for primer design all originated from oral cavity and were therefore classified as oral squamous cell carcinoma (OSCC), a subset of HNSCC, a first validation was performed on 17 OSCC and 46 control tissue samples, excluding those which were used in nanopore sequencing.

**Fig. 6:**
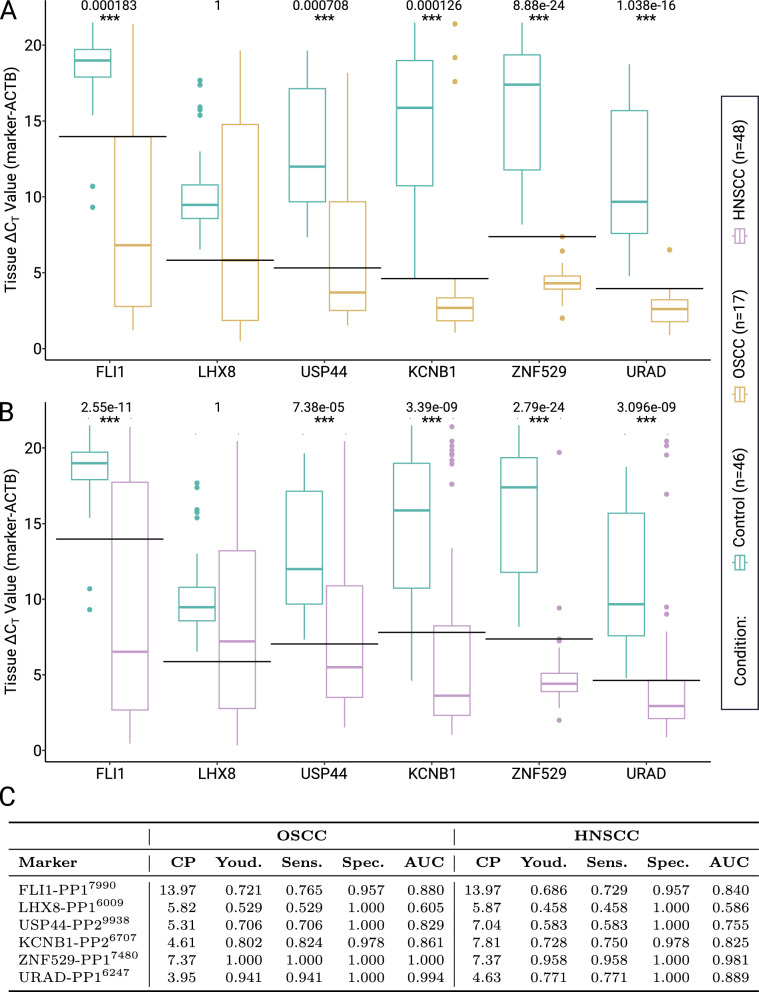
For each marker, an MSP assay was performed **A** on 17 OSCC and 46 control tissue samples, and **B** on 48 HNSCC (including the 17 OSCCs) and the same 46 control tissue samples. All markers except LHX8 show significant differences in the $\Delta$C$_T$ values between control and OSCC samples as well as between control and HNSCC samples. Horizontal lines indicate optimal cut-points. Numbers on top are adjusted *p* values, three stars indicate a value below 0.01. **C** The marker ZNF529 outperforms the other markers with Youden’s J and AUC above 0.95 for both OSCC and HNSCC samples. Except for LHX8 in the HNSCC samples, all markers perform well with a Youden’s J above 0.5. Sensitivity (Sens.), Specificity (Spec.), and AUC were calculated for the optimal cutpoint (CP), which maximized Youden’s J statistic

We confirm the diagnostic potential for five of the six markers in additional OSCC samples, as shown in Fig. 6A. All primer pairs, except LHX8-PP1$^{6009}$, show significant differences in the $\Delta$C$_T$ values between OSCC and control tissue samples, confirmed by highly significant adjusted *p* values. The marker ZNF529-PP1$^{7480}$ allows a nearly perfect classification into OSCC and control (Bonferroni corrected *p* value of 2.79$\cdot$10$^{-22}$), with a sensitivity, specificity, and AUC of 1, as shown in Fig. 6C.

The potential markers FLI1-PP1$^{7990}$, USP44-PP2$^{9938}$, KCNB1-PP2$^{6707}$, and URAD-PP1$^{6247}$ have a AUC above 0.8, and thus, all show good diagnostic accuracy. Generally, this type of analysis indicates a marker’s diagnostic potential (e.g., an AUC of 0.5754 for LHX8-PP1$^{6009}$ suggests it may not be suitable as a diagnostic marker) [48]. While USP44-PP2$^{9938}$ had the highest score in the *in silico* prediction and performed well in MSP of the sequenced samples, as given in Table 3, it was outperformed by ZNF529-PP1$^{7480}$ and URAD-PP1$^{6247}$ in the additional OSCC samples in terms of both Youden’s J and AUC.

***Primers selected from OSCC show strong diagnostic performance across 48 clinical HNSCC samples***

Although the primers were initially designed and tested using OSCC samples, a subset of HNSCC, they demonstrated robust diagnostic potential across a broader cohort of 48 clinical HNSCC tissue samples. This cross-applicability underscores the broader relevance and potential clinical utility of the selected methylation markers.

The transferability of the results from an OSCC subset of HNSCC samples to HNSCC tissue samples from different localizations was convincingly demonstrated in an MSP assay performed on 48 HNSCC samples (including the 17 OSCC samples analyzed in Fig. 6A) and the same 46 control samples as before, as shown in Fig. 6B. The results for the 48 HNSCC samples closely mirror those from the 17 OSCC samples, highlighting the robustness of the markers in an even more general context: For the primer pairs FLI1-PP1$^{7990}$, USP44-PP2$^{9938}$, KCNB1-PP2$^{6707}$, ZNF529-PP1$^{7480}$, and URAD-PP1$^{6247}$ , the MSP results in significantly different $\Delta$C$_T$ values for control and HNSCC tissues with adjusted *p* values below 0.01. Consistently with the OSCC samples, only the primer pair LHX8-PP1$^{6009}$ does not show significant differences between the two conditions. Most prominent are the markers ZNF529-PP1$^{7480}$ and URAD-PP1$^{6247}$, which both have low $\Delta$C$_T$ values with rather low variance (ZNF529-PP1$^{7480}$: mean 4.94, standard deviation 2.55; URAD-PP1$^{6247}$: mean 4.63, standard deviation 4.89) for the HNSCC samples. The marker FLI1-PP1$^{7990}$ stands out by having high $\Delta$C$_T$ values with low variance for the control samples (mean 18.42, standard deviation 2.31). These results are specifically striking, as the primers were predicted and designed based on three male-only HNSCC samples from oral cavity, while the samples used for Fig. 6B are highly diverse in sex, age, tumor localization and HPV status, as given in Table 1.

Thus, the markers FLI1-PP1$^{7990}$, KCNB1-PP2$^{6707}$, ZNF529-PP1$^{7480}$, and URAD-PP1$^{6247}$ show a high diagnostic performance with an AUC above 0.8, which classifies them as markers with very good diagnostic accuracy, as described by Šimundić [48]. Additionally, these four markers are characterized by a sensitivity above 0.7 combined with a specificity above 0.95, resulting in an Youden’s J statistic above 0.68, as shown in Fig. 6C.

In summary, the performance of an MSP assay with the described primers on 48 HNSCC and 46 control samples confirmed the generalizability of the predicted marker regions, as shown in Fig. 6B and C.

## Conclusions

Although the existence of cancer-associated DNA methylations has been known for over 25 years [1], their translation into clinical (IVD) tests using DNA methylation for cancer screening is still a long way off [4]. In this work, we presented a workflow for the prediction of DNA methylation-specific PCR biomarkers by nanopore sequencing. We showed that nanopore sequencing of three control and three tumor samples is sufficient for the prediction and design of MSP primers using the diffMONT tool [28], resulting in an AUC between 0.586 and 0.981 in 48 and 46 HNSCC and control samples, respectively. Finding new and appropriate candidate biomarkers for cancer or other disease entities without diagnostic tests is a prerequisite for developing new IVD assays. This procedure can be very time- and resource-consuming, as often only a small proportion of candidate regions identified with NGS or Array technology show good analytical and especially diagnostic results in test cohorts. This leads to high costs during marker identification (laboratory staff, chemicals, test samples) and therefore a high economic uncertainty for the inventors. With our technology using ONT sequencing with adaptive sampling followed by evaluation using the diffMONT tool, we had very promising results for 5 marker regions, coming from 20 regions being evaluated with MSP in wet lab experiments. This is a 25% hit rate which may even be improved, as we only used 6 MSPs in the clinical testing set due to resource considerations, even though 10 MSPs showed good analytical performance on discriminating methylated and non-methylated DNA.

While this study is not intended to present a final assay for clinical use, it serves as a valuable tool to guide the design and testing of similar assays on larger cohorts, potentially involving several hundred individuals. A higher number of patients in nanopore sequencing and higher coverage would improve the performance of the resulting MSP assay. The next steps, building on the five identified MSP marker regions presented in this work, would be to combine the presented primer regions into a multiplexed MSP assay to reduce MSP reactions per sample and to perform the assay in a larger cohort. In addition, a final screening assay would preferably be based on non-invasive material like saliva or smear test. Altogether, this DNA methylation-based workflow demonstrates the potential to design clinically relevant and highly adaptable MSP assays—even from as few as six tissue samples—paving the way for broader applications in cancer and disease diagnostics.

## Additional file

Supplementary file 1 (bed 610 KB)

Supplementary file 2 (pdf 2628 KB)

## Data Availability

The raw Nanopore sequencing data generated from human samples in this study cannot be made publicly available due to ethical and legal restrictions. Primer and probe sequences are available upon request. All further data generated or analyzed during this study are included in this published article and its information files.

## Footnotes

1 https://github.com/epi2me-labs/modbam2bed.
