# Supplementary material for: Nanopore sequencing-derived methylation biomarker prediction for methylation-specific PCR in patients with head and neck squamous cell carcinoma
Source: Clin Epigenetics. 2025 Sep 13;17:149. doi: 10.1186/s13148-025-01960-7 (PMC12433006; doi:10.1186/s13148-025-01960-7)
Supplement: Supplementary file 2 — (pdf 2628 KB) [file 13148_2025_1960_MOESM2_ESM.pdf]

Supplement

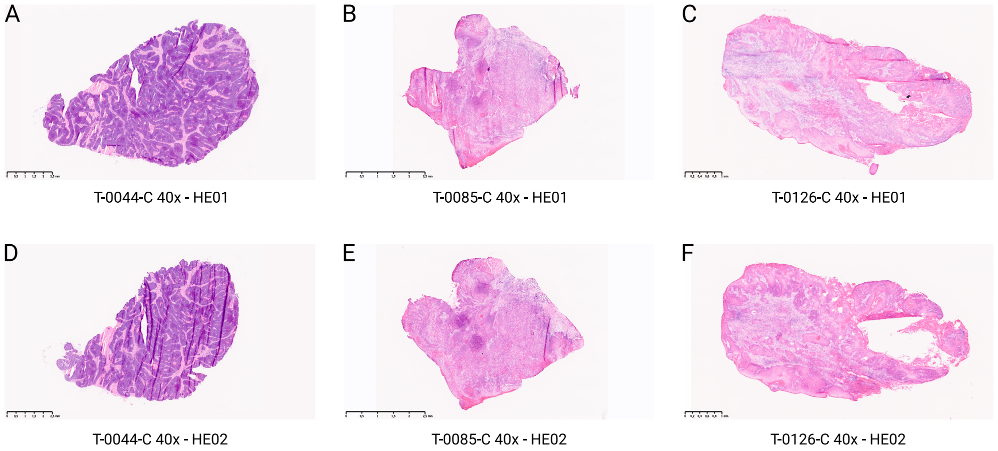

Fig. S1 The first (HE01) and last (HE12) of the twelve 10 µm tissue sections, were H&E stained to assess tumor cell content. Exemplarily the three HNSCC samples T-0044-C, T-0085-C, and T-0126-C are shown. These samples were nanopore sequenced.

Control Samples				
patientID	Sex	Localization	Age	HPV
T-0025-N	male	Uvula	59	neg.
T-0026-N	male	Uvula	58	neg.
T-0027-N	male	Tonsil	23	-
T-0028-N	male	Tonsil	58	-
T-0041-N	female	Tonsil	23	-
T-0043-N	male	Tonsil	24	-
T-0045-N	male	Uvula	22	neg.
T-0061-N	female	Vallecula	76	-
T-0062-N	male	Uvula	43	-
T-0063-N	male	Tonsil	24	-
T-0064-N	female	Tonsil	23	-
T-0066-N	female	Tonsil	18	-
T-0067-N	female	Tonsil	18	-
T-0068-N	male	Larynx	60	-
T-0069-N	male	Uvula	41	-
T-0070-N	female	Tonsil	56	-
T-0072-N	male	Uvula	44	-
T-0073-N	male	Vallecula	50	-
T-0078-N	male	Uvula	51	-
T-0081-N	male	Uvula	46	-
T-0082-N	male	Uvula	58	-
T-0087-N	male	Tongue	63	-
T-0091-N	male	Glottis	69	-
T-0094-N	male	Uvula	38	-
T-0097-N	female	Tongue	68	-
T-0098-N	female	Tongue	59	-
T-0099-N	male	Uvula	59	neg.
T-0100-N	male	Uvula	32	-
T-0101-N	male	Larynx	52	-
T-0105-N	female	Vallecula	68	neg.
T-0108-N	male	Larynx	79	-
T-0109-N	male	Tonsil	68	-
T-0111-N	male	Buccal mucosa	64	-
T-0116-N	female	Buccal mucosa	47	-
T-0120-N	male	Tonsil	39	-
T-0123-N	male	Uvula	59	-
T-0131-N	male	Tonsil	18	-
T-0133-N	female	Uvula	59	neg.
T-0135-N	male	Palatal arch	31	-
T-0136-N	male	Uvula	54	-
T-0137-N	female	Tonsil	27	-
T-0140-N	male	Tonsil	29	-
T-0144-N	female	Tonsil	38	-
T-0146-N	female	Tonsil	27	-
T-0147-N	male	Uvula	33	-
T-0148-N	male	Uvula	31	-
T-0172-N	female	Tonsil	36	neg.
T-0173-N	male	Tonsil	30	neg.
T-0174-N	male	Tonsil	28	neg.

Patient Samples								
patientID	Sex	Diagnosis	Age	T	N	M	HPV	TZ
T-0001-C	male	TC	74	4	0	0	neg.	55
T-0002-C	male	HPC	75	2	1	0	neg.	30
T-0003-C	male	HPC	55	3	0	0	neg.	50
T-0004-C	male	HPC	68	4	0	0	neg.	55
T-0005-C	male	TC	60	1	2	X	pos.	15
T-0006-C	male	HPC	62	4	2	0	neg.	35
T-0008-C	male	TC	52	4	1	0	pos.	15
T-0009-C	female	TC	72	2	2	0	pos.	25
T-0010-C	male	LC	51	1	0	0	neg.	70
T-0011-C	male	HPC	61	2	X	0	neg.	80
T-0013-C	male	OSCC	61	4	0	0	neg.	10
T-0014-C	male	LC	59	3	0	0	neg.	70
T-0016-C	male	LC	54	2	0	0	neg.	10
T-0017-C	male	OSCC	64	4	2	0	neg.	80
T-0018-C	female	LC	68	3	2	0	neg.	35
T-0020-C	male	HPC	56	3	3	0	neg.	5
T-0021-C	female	OSCC	50	1	0	X	neg.	10
T-0023-C	female	OSCC	71	3	3	X	neg.	5
T-0024-C	male	LC	61	4	0	0	neg.	30
T-0032-C	female	OSCC	34	3	2	X	neg.	20
T-0033-C	female	LC	64	4	2	1	neg.	50
T-0035-C	male	OSCC	77	2	0	X	neg.	60
T-0040-C	male	LC	64	4	0	X	neg.	25
T-0042-C	male	LC	61	4	0	X	neg.	70
T-0044-C	male	OSCC	53	3	0	X	pos.	80
T-0047-C	male	OSCC	64	4	3	0	neg.	50
T-0049-C	male	OSCC	54	3	0	0	neg.	40
T-0051-C	male	OSCC	67	2	3	0	pos.	50
T-0054-C	male	HPC	52	3	3	0	neg.	50
T-0055-C	female	LC	77	2	1	0	neg.	5
T-0060-C	male	OSCC	58	2	3	0	neg.	20
T-0071-C	male	LC	61	1	0	0	neg.	30
T-0075-C	male	TC	55	4	1	0	neg.	10
T-0077-C	male	OSCC	82	4	2	0	neg.	80
T-0080-C	male	HPC	61	4	1	X	neg.	80
T-0085-C	male	OSCC	75	4	1	0	neg.	80
T-0106-C	male	OSCC	70	3	1	0	neg.	70
T-0107-N	female	TC	63	2	1	X	pos.	15
T-0117-C	female	OSCC	53	2	0	0	neg.	60
T-0119-N	male	OPC	66	1	0	X	neg.	75
T-0122-C	male	HPC	77	3	3	0	neg.	20
T-0126-C	male	OSCC	61	4	0	0	neg.	70
T-0127-C	female	OPC	66	1	0	0	neg.	90
T-0128-C	male	LC	69	3	1	1	neg.	80
T-0129-C	male	OSCC	46	4	1	0	pos.	-
T-0141-C	male	OSCC	52	4	2	0	neg.	80
T-0149-C	male	TC	66	2	1	1	pos.	30
T-0150-C	male	HPC	66	3	0	0	neg.	90
T-0154-C	male	OSCC	79	1	0	0	neg.	40
T-0158-C	male	OSCC	54	2	2	0	neg.	25
T-0162-C	male	OPC	53	3	2	X	pos.	10

Table S1 Clinical data for all analyzed control and tumor samples. Marked in gray are the samples, which were nanopore sequenced.
Age is given in years; Tumor Diagnoses are abbreviated as follows: OSCC - Oral squamous cell carcinoma, OPC - Oropharyngeal carcinoma, TC - Tonsil carcinoma, LC - Laryngeal carcinoma, HPC - Hypopharyngeal carcinoma; TNM – cancer stage based on the TNM (T - tumor size, N - lymph nodes, M - metastasis) cancer staging system; Tumor cell percentage (TZ) is given in percent.

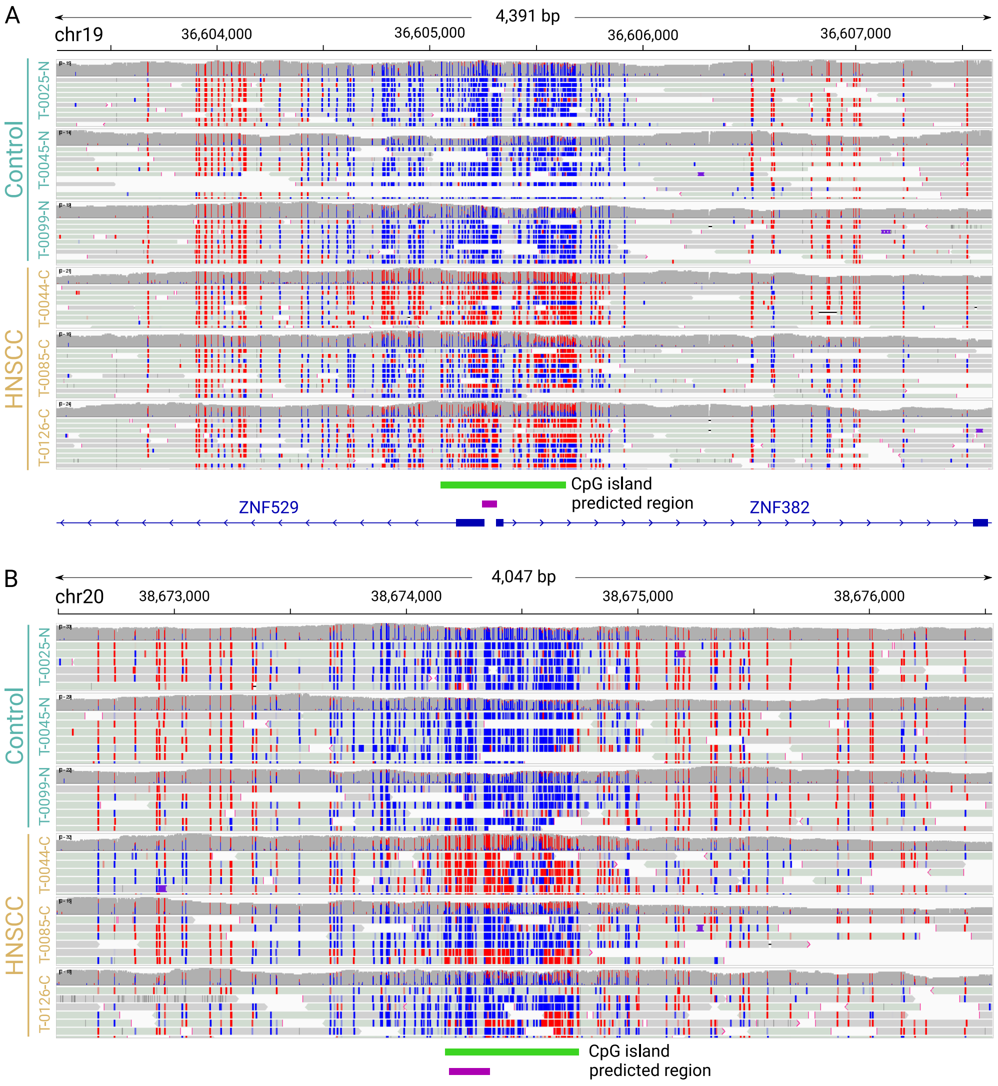

Fig. S2 *In silico* predicted differentially methylated regions all overlap with CpG islands but occur in different genomic contexts. For three exemplary regions the genetic context is shown. **(A)** This predicted region is located on chromosome 19, and overlaps a CpG island as well as the 5' ends of both ZNF529 and ZNF382. **(B)** Another predicted region is overlapping a CpG island, but no gene or transcript is annotated in close proximity.

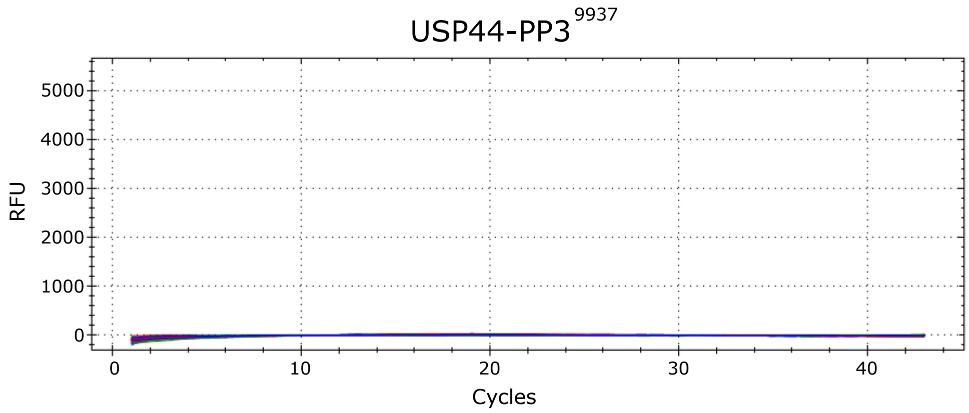

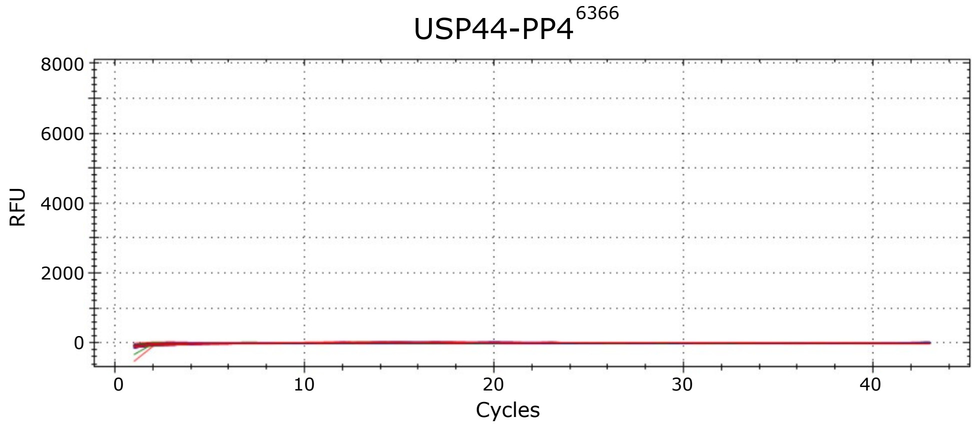

Fig. S3 For USP44-PP3^{9937} and USP44-PP4^{6366} no probe signal was detectable for the primer probe combinations during gradient PCR in any tested temperature. USP44-PP3^{9937} was tested for 70°C, 69.5°C, 68.4°C, 66.4°C, 64°C, 62°C, 60.7°C, and 60°C. USP44-PP4^{6366} was tested for 69°C, 68.4°C, 67.2°C, 65°C, 62.3°C, 60.2°C, 58.7°C, and 58°C.

Table S2 Out of 284 MSP prediction (with a score ¿6,000), 23 distinct highest-scored regions were defined. For each of these, the `diffMONT` results are listed in the table. One region is located on an unassembled contig (chr1_fix = chr1_KZ208906v1_fix). Information for both primer (Primer1, Primer2) and for the complete Amplicon region covered by both primers (Amplicon) is given. Str. - Strand for which region is predicted; #C – number of differentially methylated CpGs in the predicted primer region; Len. – region length of either predicted primer or complete amplicon; LOC* = LOC105373629

		Primer1			Primer2			Amplicon		
Chr	Str.	#C	Len.	Score	#C	Len.	Score	Len.	Score	Annotated Genes
chr12	-	7	18	4489.31	8	21	5448.51	204	9937.82	USP44
chr11	-	4	17	2573.43	6	17	5416.70	206	7990.13	SENCR; FLI1
chr2	-	7	23	4416.49	5	24	3237.52	98	7654.01	LINC02579
chr19	-	6	22	4233.27	5	22	3247.22	70	7480.49	ZNF529
chr20	+	6	19	4693.25	4	14	2733.76	98	7427.01	
chr8	-	5	18	4318.39	3	22	2648.76	65	6967.15	OPLAH
chr17	-	5	20	4407.25	3	17	2523.68	188	6930.93	PER1
chr17	-	6	24	3474.83	5	23	3314.56	130	6789.39	RAI1
chr20	+	3	4	2517.92	5	18	4188.76	177	6706.68	
chr16	-	4	22	3831.44	3	23	2762.34	305	6593.78	KREMEN2
chr4	+	5	20	3247.82	6	24	3169.20	260	6417.02	BEND4
chr12	-	6	23	3936.36	5	21	2429.79	72	6366.15	
chr2	-	5	22	3032.12	6	24	3250.81	167	6282.93	EMX1
chr13	+	3	16	2369.91	5	21	3876.66	101	6246.57	URAD
chr11	+	5	22	3234.94	5	23	3008.88	93	6243.82	TSPAN4
chr1_fix	+	3	23	2920.26	4	14	3289.96	137	6210.22	RHOU, DUSP5P1
chr19	-	6	23	2999.13	6	19	3186.05	381	6185.18	MATK
chr8	-	4	22	3627.53	3	16	2503.59	78	6131.12	
chr2	+	5	21	3272.55	4	17	2840.62	154	6113.17	
chr3	+	4	13	3130.37	4	19	2982.22	206	6112.59	FAM43A
chr8	+	6	21	3468.97	5	22	2570.75	235	6039.72	BOP1, SCX
chr2	-	5	22	2677.37	6	23	3354.68	88	6032.05	TMEM163, LOC*
chr1	+	4	12	2711.46	4	15	3297.46	110	6008.92	LHX8

Table S3 Overview of the designed primers and probes and their performance in different PCR runs. Chr. – Chromosome; Temp. – Annealing Temperature; PC – Positive Control (fully methylated DNA); NC – Negative Control (fully unmethylated DNA); NTC – Non-template Control (Water); For all PCR runs the C_T values are shown. For the gradient PCR, only data for the optimal annealing temperature is shown. For all PCR runs 43 cycles were run, if no signal was detected in 43 cycles "N/A" is noted in the table. Dashes indicate that no PCR was performed.

Designed Amplicon				Gradient PCR - Primers				Dilution Series PCR – Primer Pairs					Dilution Series PCR – Primer Probe Combination					
Name	Chr.	Score *in silico*	Length [bp]	Temp. [°C]	PC 10 ng	NC 50 ng	NTC	Temp. [°C]	PC 10 ng	PC 1 ng	NC 50 ng	NTC	Temp. [°C]	PC 10 ng	PC 1 ng	PC 0.1 ng	NC 50 ng	NTC
FLI1-PP2	chr11	7990.13	146	55.0	29.28	36.11	36.18	-	-	-	-	-	-	-	-	-	-	-
PER1-PP1	chr17	6930.93	195	55.0	25.23	40.59	N/A	-	-	-	-	-	-	-	-	-	-	-
USP44-PP1	chr12	9937.82	362	55 - 68	N/A	N/A	N/A	-	-	-	-	-	-	-	-	-	-	
LINC02579-PP1	chr2	7654.01	89	65.8	33.96	N/A	N/A	-	-	-	-	-	-	-	-	-	-	
RAI1-PP1	chr17	6789.39	157	60.1	27.24	N/A	N/A	60.0	28.03	31.54	N/A	N/A	-	-	-	-	-	-
USP44-PP3	chr12	9937.82	128	57.6	29.38	N/A	N/A	58.0	28.21	31.51	N/A	N/A	-	-	-	-	-	-
USP44-PP4	chr12	6366.15	72	60.1	27.57	N/A	N/A	60.0	31.39	34.66	N/A	N/A	-	-	-	-	-	-
FAM43A-PP1	chr3	6112.59	210	60.1	27.33	N/A	N/A	60.0	27.97	31.41	N/A	N/A	62	28.01	32.07	N/A	N/A	N/A
KREMEN2-PP1	chr16	6593.78	295	55.9	29.64	N/A	N/A	56.0	31.51	34.67	N/A	N/A	59	31.54	35.60	N/A	N/A	N/A
BEND4-PP1	chr4	6417.02	252	60.1	29.40	N/A	N/A	60.0	30.13	33.47	N/A	N/A	61	31.97	35.76	40.44	N/A	N/A
KCNB1-PP1	chr20	7427.01	117	65.8	28.18	N/A	N/A	65.0	28.18	-	N/A	N/A	61	27.43	31.26	34.73	N/A	N/A
SMDP5-PP1	chr8	6131.12	83	55.9	26.03	N/A	N/A	58.0	26.58	29.90	N/A	N/A	60	27.42	31.10	34.79	N/A	N/A
RAI1-PP2	chr17	6789.39	152	60.1	26.76	N/A	N/A	60.0	27.43	31.13	N/A	N/A	61	27.75	31.50	36.09	N/A	N/A
EMX1-PP1	chr2	6282.93	166	60.1	26.67	N/A	N/A	60.0	27.31	30.89	N/A	N/A	61	26.60	29.93	33.59	N/A	N/A
KCNB1-PP2	chr20	6706.68	181	60.1	28.32	N/A	N/A	60.0	29.15	32.50	N/A	N/A	61	29.96	33.27	37.33	N/A	N/A
ZNF529-PP1	chr19	7480.49	390	60.1	30.19	N/A	N/A	60.0	30.70	34.34	N/A	N/A	61	31.56	35.68	38.63	N/A	N/A
LHX8-PP1	chr1	6008.92	114	57.6	27.54	N/A	N/A	58.0	27.31	31.06	N/A	N/A	59	29.48	33.12	36.59	N/A	N/A
URAD-PP1	chr13	6246.57	106	57.6	34.74	N/A	N/A	58.0	36.66	41.02	N/A	N/A	61	33.38	37.34	39.34	N/A	N/A
FLI1-PP1	chr11	7990.13	112	55.9	27.15	N/A	N/A	56.0	28.66	31.93	N/A	N/A	59	30.05	33.53	36.91	N/A	N/A
USP44-PP2	chr12	9937.82	265	63.2	31.56	N/A	N/A	65.0	31.44	-	N/A	N/A	60	29.24	32.53	37.46	N/A	N/A
